# Role of Oxidative Stress in Pseudoexfoliation Syndrome and Pseudoexfoliation Glaucoma

**DOI:** 10.4274/tjo.galenos.2018.10734

**Published:** 2019-04-30

**Authors:** Yasemin Aydın Yaz, Nilgün Yıldırım, Yetkin Yaz, Neslihan Tekin, Mine İnal, Fezan Mutlu Şahin

**Affiliations:** 1 Eskişehir State Hospital, Ophthalmology Clinic, Eskişehir, Turkey; 2 Eskişehir Osmangazi University Faculty of Medicine, Department of Ophthalmology, Eskişehir, Turkey; 3 Eskişehir Yunus Emre State Hospital, Ophthalmology Clinic, Eskişehir, Turkey; 4 Aksaray University Faculty of Arts and Sciences, Department of Biotechnology and Molecular Biology, Aksaray, Turkey; 5 Eskişehir Osmangazi University Faculty of Medicine, Department of Biochemistry, Eskişehir Turkey; 6 Eskişehir Osmangazi University Faculty of Medicine, Department of Biostatistics, Eskişehir, Turkey

**Keywords:** Pseudoexfoliation, glaucoma, oxidative stress

## Abstract

**Objectives:**

To investigate the role of oxidative stress on pseudoexfoliation formation and progression from pseudoexfoliation syndrome (XFS) to pseudoexfoliation glaucoma (XFG).

**Materials and Methods:**

This study investigated oxidative stress biomarkers in blood samples from 58 patients with XFG, 47 patients with XFS, and 134 healthy age- and sex-matched controls.

**Results:**

The highest serum malondialdehyde (MDA) levels were measured in XFG patients (p<0.001), and MDA level was higher in XFS patients than controls (p<0.001). Superoxide dismutase (SOD) and catalase (CAT) enzyme activities were significantly lower in XFS and XFG patients than in the control group, whereas a significant increase was observed in glutathione (GSH) levels (p<0.001 for all). However, levels of these three biomarkers did not differ significantly between XFS and XFG patients (p=0.188, p=0.185, and p=0.733, respectively). Nitric oxide (NO) concentration was significantly lower in XFG patients compared to XFS patients and controls (p<0.001) but did not differ between XFS patients and controls (p=0.476).

**Conclusion:**

Elevated MDA levels suggest that lipid peroxidation is important in XFS and XFG development and progression from XFS to XFG. In addition, reduction in SOD and CAT enzyme activities is considered a deficiency in the enzymatic antioxidant protection system. Furthermore, GSH values may be evaluated as a compensatory response to oxidative stress in XFS and XFG. Alterations in NO indicate the role of a vascular regulatory factor in the progression from XFS to glaucoma.

## Introduction

Pseudoexfoliation syndrome (XFS) is an age-related extracellular matrix disorder that is associated with the excessive production and accumulation of abnormal fibrillar material in intra- and extraocular tissues. In all populations, the frequency of XFS increases with age and the incidence of the syndrome doubles every decade.^1^ The accumulation of abnormal fibrillar aggregates in the outflow pathways leads to an increase in outflow resistance and intraocular pressure.^2^ XFS is a significant cause of chronic open-angle glaucoma and can predispose individuals to a broad spectrum of intraocular and surgical complications. Furthermore, XFS is not only an ocular disease but is also considered to be a systemic disorder due to the accumulation of pseudoexfoliative material (XFM) in visceral organs such as the heart, lung, gallbladder, kidney, and cerebral meninges.^3^

It is well known that oxidative stress plays an important role in XFS and other age-related disorders such as cataract and age-related macular degeneration.^4^ Oxidative stress is defined as an imbalance between oxidants and antioxidants or an increase in the intracellular concentrations of reactive oxygen species (ROS) over physiological values. ROS such as hydrogen peroxide (H_2_O_2_), hydroxyl radical (OH), and nitric oxide (NO) incorporate into proteins, lipids, carbohydrates, and nucleic acids, then promote DNA damage and cellular injury.^5^ On the other hand, antioxidant defense systems can protect cells from the detrimental effects of ROS. Oxidative stress can induce an increase or decrease in the antioxidant defense system as a protective response or due to the ROS effect, respectively.^6^

Oxidative stress plays a key role in the pathogenesis of XFS and glaucoma.^7^ Different ROS and antioxidants have been investigated in serum and aqueous samples in previous studies. Total oxidative stress (TOS)^8^, malondialdehyde (MDA)^9^, 8-hydroxydeoxyguanosin (8-OHdG)^10^, protein carbonyl (PC)^9^, and nitric oxide (NO)^9^ were measured as oxidant markers, and total antioxidant status (TAS)^11^, superoxide dismutase (SOD)^9,12^, glutathione peroxidase (GPx)^12^, catalase (CAT)^12^, vitamin C^12^, paraoxonase^8,13^, and arylesterase^8^ were measured as antioxidant markers in different studies.

Despite the recent studies, the exact pathogenesis of XFS and the progression from XFS to glaucoma remain unclear. In this study, we aimed to investigate the effect of oxidative stress on the development of XFS and progression from XFS to pseudoexfoliation glaucoma (XFG). Therefore, we measured the activity of the SOD and CAT enzymes (enzymatic antioxidants), MDA (an end product of lipid peroxidation), NO (a marker of nitrosative stress and vascular function), and GSH (a primary endogenous antioxidant) as oxidative stress biomarkers in patients with XFG, XFS, and healthy control subjects.

## Materials and Methods

### Study Population

The study population comprised 239 individuals, including 58 patients with XFG, 47 patients with XFS, and 134 healthy age- and sex-matched controls. Written informed consent was obtained from all participants. The study was approved by the ethics review board of the Eskişehir Osmangazi University Faculty of Medicine and adhered to the tenets of the Declaration of Helsinki.

All subjects underwent a standardized detailed ophthalmic examination that included assessments of refraction, visual acuity, and intraocular pressure (Goldmann applanation tonometry) as well as fundus and anterior segment biomicroscopy examinations. XFG was defined as the presence of XFM on the anterior lens capsule or pupillary margin, elevated intraocular pressure (IOP) (≥21 mmHg), glaucomatous optic disc changes (vertical cup-to-disc ratio [C/D] ≥0.5, C/D asymmetry ≥0.2), and characteristic visual field defects in computed perimetry (Zeiss Humphrey visual field analyzer white on white 30-2 threshold program). Patients who had XFM in the anterior lens capsule and pupillary margin but whose IOP, optic disc, and visual field findings were within normal limits were defined as having XFS. The control group was matched with the patient cohorts based on age and sex and underwent a standardized detailed ophthalmic examination. The controls did not exhibit XFM, had IOP within the normal range (<21 mmHg), and had no glaucomatous optic disc damage. All participants were questioned about systemic diseases (diabetes, hypertension, thyroid and rheumatic diseases) and drug usage. We excluded patients with ophthalmic diseases (e.g., uveitis, angle closure glaucoma, pigment dispersion syndrome, trauma, progressive retinal disease), smokers, and patients with uncontrolled major systemic diseases.

### Sample Preparation

Blood samples were collected in two different tubes. The first tube was centrifuged at 3,500 x g for 10 minutes to separate the serum and was used for the determination of NO, MDA, and CAT concentrations. The second tube, which included EDTA, was used for the measurement of GSH and SOD. The samples were immediately centrifuged at 1,500 x g for 5 min, and the plasma was separated. After separating the plasma, the erythrocytes were washed three times with saline and erythrocyte packets were prepared. Erythrocyte hemolysates were then prepared and stored at -80 °C until GSH and SOD measurement.

### Determination of MDA Level

Serum lipid peroxidation was estimated based on the measurement of malondialdehyde reacted with thiobarbituric acid (TBA), according to the method described by Ohkawa et al.^14^ Absorbance was measured at 532 nm. MDA levels were presented in nmol/L.

### Determination of SOD Activity

Erythrocyte SOD activity was assayed spectrophotometrically, according to the method described by Winterbourn et al.^15^ This assay is based on the inhibitory effect of SOD on the reaction in which superoxide anion reduces nitroblue tetrazolium (NBT). Absorbance was measured at 560 nm. SOD activity was presented in U/Hb.

### Determination of CAT Activity

Serum CAT activity was determined according to the method described by Beutler.^16^ This method is based on the rate of hydrogen peroxide decomposition due to the activity of CAT in the examined samples. Absorbance was measured at 230 nm. CAT activity was presented in U/L.

### Determination of GSH Level

Erythrocyte GSH levels were measured spectrophotometrically according to the method described by Ellman et al.^17^ GSH is reacted with 5.5 dithiobis-2-nitrobenzoic acid (2 DTNB), resulting in the formation of a product that has a maximal absorbance at 412 nm. GSH levels were also presented in U/Hb.

### Determination of NO Level

Serum nitrite (NO_2_ˉ) and nitrate (NO_3_ˉ) were assessed as an index of NO production, based on the cadmium reduction method described by Wakid and Cortas.^18^ The samples were deproteinized, and total nitrite (nitrite + nitrate) was measured via spectrophotometry at 545 nm after the reduction of nitrate to nitrite with copperized cadmium granules. The results were presented in µmol/L.

### Statistical Analysis

Statistical analysis was performed using SPSS version 20.0 for Windows. The Shapiro-Wilk normality test was applied for continuous variables. Normally distributed variables were analyzed using a t test for independent groups and summarized using the mean and standard deviation. Non-normally distributed variables were compared using the Mann-Whitney U test and the Kruskal-Wallis test and summarized using the median and 25^th^ and 75^th^ percentiles. The Pearson chi-square test was used for categorical variables; the results were summarized using the sample size (n) and percentage (%). The comparisons of MDA, SOD, CAT, GSH, and NO between the groups were analyzed by Quade’s Rank Analysis of Covariance. Systemic disease was used as a covariant. The Dwass-Steel-Critchlow-Fligner multiple comparison method was used to determine significantly different groups. P value less than 0.05 was accepted as the level of significance.

## Results

A total of 239 individuals over 40 years of age were recruited for this study by the Eskişehir Osmangazi University Glaucoma Department as follows: 58 individuals with XFG, 47 with XFS, and 134 controls. The demographic data of the subjects are shown in Table 1.

The levels of MDA, SOD, CAT, GSH, and NO were summarized in Table 2. Patients under a treatment regimen for systemic diseases such as diabetes, hypertension, and others (e.g., cardiovascular disease, rheumatologic disease) were compared with those without systemic disease and no statistically significant difference was found (p<0.001). As a result, they were included in the study. The levels of oxidative stress markers between patients with and without systemic diseases in the different groups is shown in Table 3.

Serum MDA levels were significantly higher in XFG patients than in XFS patients or controls (p<0.001). In addition, XFS patients’ MDA levels were also higher than those of the controls (p<0.001). The SOD and CAT enzyme activities of XFS and XFG patients were significantly lower than those of the control group (p<0.001). However, no differences were observed in SOD and CAT activity between XFS and XFG patients (p=0.188 and p=0.185, respectively). GSH levels were significantly higher in XFS and XFG patients when compared to control subjects (p<0.001). Similar to the SOD and CAT activities, no significant difference was observed in GSH concentration between the XFS and XFG groups (p=0.733). In addition, the concentration of NO was significantly lower in XFG patients than in XFS patients and control subjects (p<0.001). However, NO levels did not differ between XFS patients and controls (p=0.476).

## Discussion

XFS is a multifactorial systemic disease in which genetic and environmental risk factors play a role in pathogenesis. Disturbances in the balance between ROS and antioxidant defense systems contribute to the development of XFS. There is increasing evidence that the oxidant-antioxidant balance is disrupted in XFS, not only in the anterior segment, but throughout the body. Intraocular secretion of XFM is closely related to aqueous circulation; therefore, examination of aqueous humour and lens epithelial cell composition in patients with XFS may reveal important pathogenetic factors.^19^ In recent years, *LOXL1* single nucleotide polymorphisms (SNPs) have been identified as a risk factor for XFS. Despite the association between *LOXL1* SNPs and XFS, the high frequency of these SNPs in the non-XFS population indicates that different factors may play a role in the development of XFS.^20^ Furthermore, results from several studies show that local production of growth factors, especially TGFβ1, seems to play an important role in XFS. TGFβ1 induces the expression of *LOXL1* and other extracellular matrix proteins in XFM.^21^ Based on evidence of epigenetic correlations with XFG, metabolic, physical and environmental conditions would affect the biological functions of XFS-related proteins by changing their expression, secretion, and conformation.^22^ Despite the effects of oxidative stress and genetic and epigenetic factors on XFS development, the exact pathogenesis of XFS remains unclear.

MDA is the end-product of polyunsaturated fatty acid peroxidation and reflects free radical damage caused by lipid peroxidation. It also seems to be a good biomarker for evaluating oxidative stress in serum.^7^ In the current study, we observed differences in serum MDA levels between the study groups. The highest values were observed in the XFG group, and the MDA levels of XFS patients were higher than those of the control group. High MDA levels suggest that the effects of oxidative stress play a role in XFM formation and in XFG development. In previous studies, similar results were found for MDA levels. Yağci et al.^23^ and Yılmaz et al.^24^ found elevated serum MDA levels in XFS patients in comparison to healthy controls. Gartaganis et al.^25^ reported a 2.5-fold increase in MDA levels in lens epithelial cells of patients with XFS in comparison to lens epithelial cells from non-XFS patients. A study performed by Engin at al.^26^ demonstrated that MDA levels in glaucoma patients with XFS were higher than in other glaucoma patients and the control group. Another study performed by Faschinger et al.^27^ reported high levels of thiobarbituric acid-reacting substances (TBARS), which are major breakdown products of lipid peroxides, in aqueous samples from primary open angle glaucoma (POAG) patients and in serum samples from non-XFS cataract patients. However, no significant differences were observed between the groups. Similarly, Ocakoglu et al.^28^ found the MDA levels in POAG patients to be twice those of the control group. In contrast, Tetikoğlu et al.^29^ found no difference between the control and XFS groups. In the same study, the mean serum MDA levels in the XFS and XFG groups were comparable, with no statistically significant difference.

SOD and CAT are key antioxidant enzymes in the metabolism of ROS, and the levels of these enzymes reflect the oxidative stress status and oxidative stress response of the organism. SOD specifically converts superoxide radicals to hydrogen peroxide and oxygen. In our study, the SOD and CAT enzyme activities of XFS and XFG patients were significantly lower than the control group, while no significant differences were observed between XFS and XFG patients. These results suggest an inadequate antioxidant enzyme response and might demonstrate a role in pseudoexfoliation development. However, the progression from XFS to XFG could not be explained with these results. Similar findings were obtained in studies performed by Yağci et al.^23^ and Engin et al.^26^ reporting decreased serum SOD levels in XFS patients in comparison to control subjects. Additionally, SOD was investigated in aqueous and lens epithelium samples in different studies. Ucakhan et al.^30^ reported an increase in SOD activity in the lens capsules of patients with XFS and cataracts. In another study in which Ferreira et al.^12^ analyzed aqueous samples, higher SOD activity was observed in XFG patients than in the POAG and cataract groups. No significant differences were found between the two glaucoma groups, but a significant increase in SOD activity was found between the glaucoma group and cataract group. Despite the decrease in SOD in serum, an increase in aqueous and lens capsules could be a protective response of the eye against oxidative stress.^7^

CAT is an antioxidant enzyme that catalyzes the decomposition of hydrogen peroxide to molecular oxygen and water. In the current study, CAT activity was significantly lower in XFS and XFG patients compared to the control group. The reduction of CAT activity observed in XFS and XFG patients was interpreted as an insufficiency of antioxidant enzymes or a decrease in enzyme levels in response to oxidative stress. Koliakos et al.^31^ found significantly lower CAT activity in both serum and aqueous samples from XFS and XFG patients compared with samples from controls. Similarly, a decrease in serum CAT activity was reported in a study performed by Zoric et al.^32^ In another study, Ferreira et al.^21^ found no significant differences in CAT activity between aqueous samples from the XFG, POAG, and cataract groups. Tetikoğlu et al.^29^ reported an insignificant decrease in serum CAT activity in pseudoexfoliative group, in contrast to our findings.

GSH is a tripeptide and the major endogenous antioxidant molecule. This molecule is involved in the cellular portion of the antioxidant defense system.^25^ In our study, the GSH levels of XFS and XFG patients were significantly higher than those of the control group, whereas no significant differences were observed between the XFS and XFG groups. The difference observed between the pseudoexfoliative and control groups was interpreted as a compensatory defense mechanism against oxidative damage. In contrast to SOD and CAT, GSH levels were higher in the pseudoexfoliation group than in the control group. SOD and CAT are enzymatic antioxidants; however, GSH is non-enzymatic and represents the first defense mechanism of the organism against oxidative stress. Our results were not consistent with those of previous studies. Gartaganis et al.^25^ found a 2.2-fold decrease in GSH levels in XFS lens epithelial cells in comparison to non-XFS lens epithelial cells. In another study performed by the same group, aqueous humor samples from individuals with XFS exhibited a decrease in GSH concentrations of up to 28%.^33^ These studies used different types of samples than our study, and their results might indicate a local response to oxidative stress.

Vascular-derived cellular mediators are important in glaucoma pathogenesis as well as IOP elevation. The vascular endothelium plays a major role in vascular homeostasis by producing these cellular mediators. Vascular microcirculation depends on the balance between vasodilation mediators (e.g., NO, prostacyclin, and hydrogen peroxide) and vasoconstrictor mediators (e.g., endothelin-1, angiotensin, and thromboxane). NO is a key molecule for vasodilation. In addition to its role as a vascular mediator, NO is also a neurotransmitter, a free radical, and an antioxidant. In vascular endothelial diseases, an increase in vascular permeability, disturbances in VEGF production, increased responses to endothelin-1, and decreased responses to NO have been observed.^34^

Ocular blood flow abnormalities play a role in the pathogenesis of glaucoma. Reduction in ocular blood flow is thought to be secondary to vascular dysregulation.^35^ NO is the major vasodilator molecule in the choroid, optic nerve, and retina; therefore, NO is a key molecule for ocular blood circulation. Vascular endothelial disease in glaucoma also affects endothelial cells in the trabecular meshwork and Schlemm’s canal, as well as in vascular endothelial cells. NO has a vasodilator effect on vascular endothelial cells, but in the trabecular meshwork, NO regulates trabecular outflow by contracting trabecular cells.^36^ Furthermore, NO facilitates aqueous outflow and causes a decrease in IOP; in contrast, endothelin-1 increases IOP. Therefore, NO and endothelin-1 are associated with IOP elevation in glaucoma through a decrease in NO production or an excessive increase in endothelin-1 secretion.^37^

In our study, we found significantly lower NO levels in XFG patients than XFS and control groups. Reduced NO levels in XFG could be a contributory factor to glaucoma development with no specific role in XFS. This is supported by previous studies which have failed to find any statistically significant differences in NO levels between subjects with XFS and controls.^38,39^ Borazan et al.^38^ evaluated VEGF and NO levels in the plasma and aqueous humor of XFS and XFG patients and found no significant differences in plasma NO levels between the XFS, XFG, and control groups. In another study performed by Altintas et al.^39^, NO levels were found to be slightly higher in XFS and XFG patients than in POAG and control patients, but the observed differences were not statistically significant. Yağci et al.^40^ found that serum nitrite levels were significantly higher in the pseudoexfoliative group than in controls. Similarly, another study performed by Erdurmus et al.^9^ reported higher NO levels in POAG and XFG patients than in controls.

### Study Limitations

Our study has several limitations. We assessed MDA, SOD, CAT, GSH, and NO levels in serum samples. Although XFS is mostly diagnosed on the basis of ophthalmic findings, it is considered a complex disease that manifests in multiple systems. Therefore, we based our methodology on revealing differences in oxidative markers in the serum sample of the patients reflecting the multisystem involvement of XFS. We included patients with systemic diseases but the results were not affected when the covariance analysis was performed. However, the presence of systemic diseases might be confounding and these patients could be excluded from the study.

## Conclusion

Finally, the results of the present study revealed a difference in MDA levels between study groups. MDA levels were the lowest in the control group, followed by the XFS and XFG groups. The observed differences in MDA levels indicate that lipid peroxidation might play a role in XFS and XFG development. SOD and CAT activities were lower and GSH levels were higher in XFS and XFG patients than in the control group. The reduction observed in SOD and CAT activities might be interpreted as a deficiency in antioxidant defense systems. The elevated levels of GSH in the pseudoexfoliative group suggests a compensatory response to oxidative stress. NO levels were lower in XFG patients than in XFS and control patients. Impaired NO levels in XFG patients might have a dual negative effect on glaucoma development and progression by affecting ocular blood flow and trabecular outflow.

In conclusion, these results suggest that oxidative stress may play a role in XFS pathogenesis. Especially lipid peroxidation and decreased antioxidant enzyme activities were found to be associated with pseudoexfoliation development. In addition, lipid peroxidation and decreased NO levels were found to be related to glaucoma progression from XFS to XFG.

## Figures and Tables

**Table 1:**
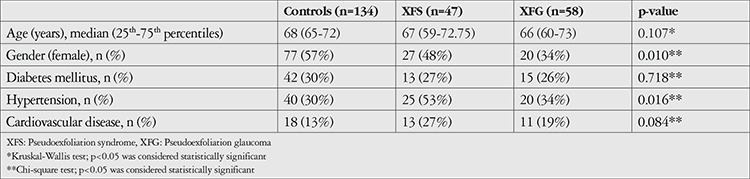
Demographic and clinical characteristics of all groups

**Table 2:**
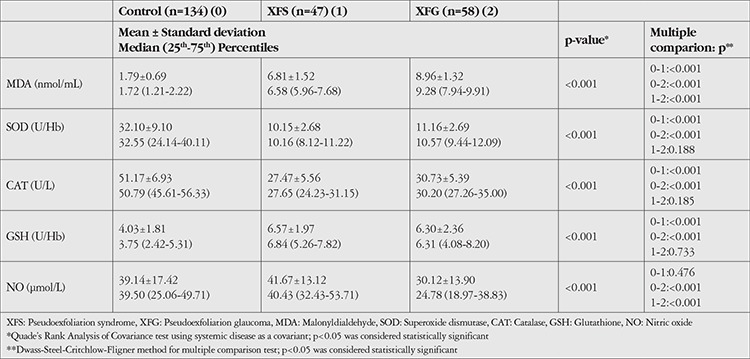
Levels of oxidative stress biomarkers in all groups

**Table 3:**
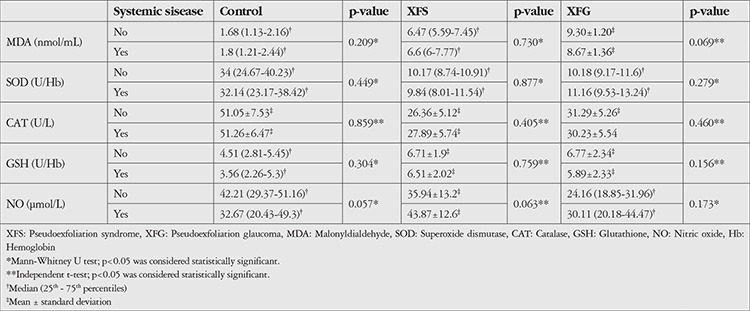
The levels of oxidative stress markers between patients with and without systemic diseases among different groups
